# Development and validation of a visual prediction model for severe acute pancreatitis: a retrospective study

**DOI:** 10.3389/fmed.2025.1564742

**Published:** 2025-07-02

**Authors:** Xiaoli Huang, Jia Xu, Xiaogang Hu, Juntao Yang, Menggang Liu

**Affiliations:** ^1^Department of Hepatobiliary and Pancreatic Surgery, The People’s Hospital of Chongqing Liang Jiang New Area, Chongqing, China; ^2^Department of Pharmacy, Chongqing Jiulongpo People’s Hospital, Chongqing, China

**Keywords:** acute pancreatitis, systemic inflammatory grade, prognosis, biomarker, prediction model

## Abstract

**Background:**

Acute pancreatitis (AP) is a common acute abdominal disease. The early identification of patients at risk of progression to severe AP (SAP) is crucial for developing effective therapeutic and nursing measures. Although many scoring systems exist for SAP risk assessment, none is widely accepted. Systemic inflammatory grade (SIG) is a novel systemic inflammation-based scoring system, but its relationship with AP, as well as the SAP risk prediction model involving SIG, has not been reported.

**Methodology:**

The demographic information, clinical data, and laboratory results of patients diagnosed with AP were collected. Baseline comparisons were made using the Wilcoxon rank-sum test, chi-square test and Fisher’s exact test. Logistic regression analyses were used to identify independent predictors of SAP; these factors were then used to establish a nomogram model. The model’s predictive efficacy and threshold values were evaluated using the receiver operating characteristic (ROC) curve and calibration curve. The decision curve analysis (DCA) and clinical impact curve (CIC) were used to further evaluate the benefit of the model.

**Results:**

Five hundred and ninety-two patients aged 18–92 years (median, 43 years) were included. In two stepwise regressions, SIG, C-reactive protein (CRP), prognostic nutritional index (PNI), and white blood cell (WBC) were all considered independent risk factors for SAP (*p* < 0.05). A nomogram prediction model was constructed using these four factors, with an area under the curve (AUC) of 0.940 (95% CI: 0.907–0.972, *p* < 0.01). The AUC-ROC for 10-fold cross-validation was 0.942 ± 0.065. The results of the Hosmer and Lemeshow goodness of fit (GoF) test (*p*-value = 0.596) and the Brier score (0.031, 95% CI 0.020–0.042), as well as the calibration curve, all demonstrated that the model exhibits good accuracy. DCA and CIC curves showed that the model provided good predictive value.

**Conclusion:**

SIG, CRP, PNI, and WBC represent promising early prognostic markers for severe acute pancreatitis (SAP). A nomogram prediction model utilizing these markers offers effective early prediction for SAP.

## Introduction

Acute pancreatitis (AP) is a prevalent acute abdominal condition that necessitates hospitalization and it is associated with severe pain and significant economic impact (1). Although most patients with AP have favorable outcomes, some progress to severe AP (SAP), which carries a mortality rate of 20–40% (2). Early identification of patients at risk of progression to SAP and adoption of better therapeutic and nursing measures could improve patient prognosis. Various scoring systems, such as the Acute Physiologic Assessment and Chronic Health Evaluation II (APACHE II), Ranson, and Bedside Index of Severity of Acute Pancreatitis (BISAP), have been employed to assess the risk of SAP. However, these systems are complex, or they depend on several parameters that are not readily available upon admission, making them impractical for immediate use upon hospital admission, particularly in hospitals with limited medical resources (3). Therefore, simpler and more effective tools for early SAP risk assessment are required.

Simple inflammation scoring systems based on blood cell counts and biochemical parameters, such as neutrophil-to-lymphocyte ratio (NLR), derived neutrophil-to-lymphocyte ratio (dNLR), platelet-to-lymphocyte ratio (PLR), prognostic nutritional index (PNI), systemic immune inflammation index (SII) and systemic inflammation response index (SIRI) have been used to predict AP severity (4–8). Although some laboratory indicators and the aforementioned scoring systems can predict the severity of AP, their clinical utility remains limited. With advancements in information technology and statistical methods, SAP prediction models based on various simple parameters have gradually attracted attention (9, 10).

Golder et al. (11) introduced systemic inflammatory grade (SIG), which combines modified Glasgow prognostic score (mGPS) and neutrophil-to-lymphocyte ratio (NLR), to assess the inflammatory response. They found that SIG was better than either the NLR or the mGPS for screening high-risk colon cancer patients who may benefit from adjuvant therapy after surgery. Another study showed that an increased SIG was strongly associated with postoperative mortality in patients with abdominal aortic aneurysms (12). Though the prognostic value of SIG have been demonstrated in chronic diseases, but its relevance in acute inflammatory diseases remains unexplored. This study aimed to evaluate the predictive value of SIG for SAP and to construct a SAP prediction model based on multiple easily measurable parameters.

## Materials and methods

### Patients

**Figure 1:**
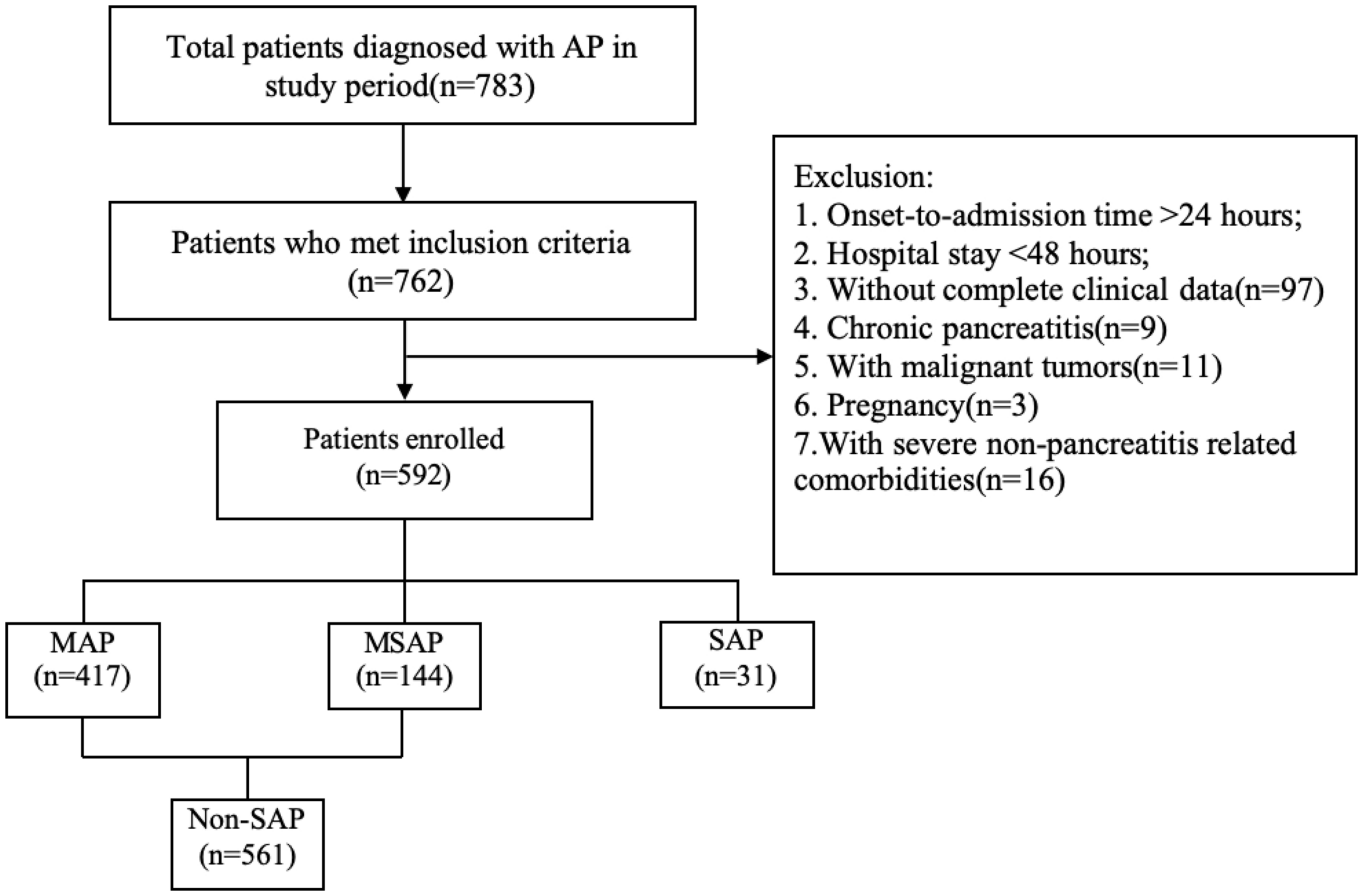
Flow chart of patient selection.

This retrospective study included patients with AP treated at our hospital between January 2021 and June 2023. All subjects met the diagnostic criteria for AP according to the revised Atlanta Classification (13). The exclusion criteria for patient selection included: time from onset of abdominal pain to admission exceeding 24 h; hospital stay shorter than 48 h; incomplete clinical data; history of chronic pancreatitis; presence of pancreatic tumors or other malignant neoplasms; pregnancy; and severe comorbidities unrelated to pancreatitis or chronic inflammatory conditions, such as autoimmune diseases and chronic organ dysfunction (Figure 1).

### Data collection

Laboratory test results from blood samples taken within 24 h of admission and clinical data were extracted from the patients’ medical records. Regarding severity, AP was categorized as mild acute pancreatitis (MAP), moderately severe acute pancreatitis (MSAP), or SAP, based on the revised Atlanta Classification. Then, MAP and MSAP were combined as “Non-SAP” for analysis. Inflammation indices were calculated according to the following methods. NLR: neutrophil count/lymphocyte count. dNLR: Neutrophil count/(total white blood cell count − neutrophil count). PLR: Platelet count/lymphocyte count. SII: platelet count * neutrophil count/lymphocyte count. SIRI: Neutrophil * monocyte count/lymphocyte count. PNI: serum albumin concentration (g/L) + 5 * lymphocyte count. The SIG was calculated using NLR and mGPS described by Golder et al. (11) (Supplementary Table 1). Continuous variables were converted into categorical variables as reported by Barrio et al. (14).

### Screening of independent risk factors for SAP and construction of prediction model

Logistic regression analysis was used to identify the risk factors of SAP, which includes a screening strategy consisting of three steps. First, univariate analysis was conducted, followed by a stepwise regression that included all features; and finally, a stepwise regression was performed on the features with *p* < 0.05 from the univariate analysis. In the stepwise analysis, the parameters were set to specify the significance level for entry = 0.05, and specify the significance level for staying in the model = 0.05. By taking the intersection of the results from the aforementioned three steps, the risk factors for SAP was obtained. Ultimately, a nomogram prediction model was created based on these factors, and DALEX package was used to explain the importance of the contribution of the features to the model.

### Evaluation of the prediction model

The model’s discrimination and calibration were evaluated using the ROC curve, and its calibration was assessed with calibration curve. Hosmer and Lemeshow goodness of fit (GoF) test as well as Brier Score were used for further quantitative assessment. A 10-fold cross-validation was employed to evaluate the stability of the model. Additionally, the model’s confusion matrix was assessed based on the maximum Youden index. Meanwhile, decision curve analysis (DCA) and clinical impact curve (CIC) were used to further evaluate the model’s clinical benefit.

### Statistical analysis

The Kolmogorov–Smirnov test was used to assess the normality of the continuous variables. Continuous variables that follow a normal distribution were expressed as mean ± standard deviation, while non-normally distributed continuous variables were presented as median and interquartile range; categorical data were shown as the number and percentage of cases. The independent sample *t*-test or Wilcoxon rank-sum test for verification was used for the comparison of measurement data between groups. Categorical data comparisons were performed using the chi-square test and Fisher’s exact test. Analyses were performed using SPSS 27.0 (IBM Corporation, Armonk, New York, United States) and R-4.3.0 (Vienna, Austria, https://www.r-project.org/), with statistical significance defined as *p* < 0.05.

### STROBE statement compliance

This observational study was conducted and reported in accordance with the Strengthening the Reporting of Observational Studies in Epidemiology (STROBE) guidelines.

## Results

### Baseline characteristics of AP patients with different severity

In total, 592 patients aged 18–92 years (median, 43 years) were included. Clinical data, laboratory examination, and inflammation scorings results for each group are presented in Table 1; Supplementary Table 2 (the cutoff values of continuous variables are shown in Supplementary Table 3). No significant differences were found in age, sex, smoking or drinking history, comorbidities, or etiology between the two groups (*p* > 0.05). Clinical outcome indicators such as local complications, hospital stay, intensive care unit (ICU) admission and mortality were correlated with AP severity. White blood cell (WBC), neutrophil counts, C-reactive protein (CRP), creatinine and blood urea nitrogen (BUN) levels were lower in the non-SAP group than in SAP groups (*p* < 0.01), whereas lymphocyte counts and serum calcium levels exhibited the reverse trend (*p* < 0.01). The NLR mGPS, SIG, SII, SIRI, and dNLR scores were significantly higher in SAP group than that in non-SAP group (*p* < 0.01), while the PNI showed the opposite trend (*p* < 0.01). The PLR did not significantly differ between the two groups (*p* > 0.05).

**Table 1:** Patient demographic characteristics and clinical data.

Variables	Non-SAP (*N* = 561)	SAP (*N* = 31)	Total (*N* = 592)	*p*-value
Demographics				
Age (≥34 years)	451 (80.39%)	29 (93.55%)	480 (81.08%)	0.113
Sex (male, %)	392 (69.88%)	19 (61.29%)	411 (69.43%)	0.418
Smoking, *n* (%)	223 (39.75%)	14 (45.16%)	237 (40.03%)	0.682
Drinking, *n* (%)	90 (16.04%)	9 (29.03%)	99 (16.72%)	0.101
Comorbidities, *n* (%)				
Hypertension	102 (18.18%)	9 (29.03%)	111 (18.75%)	0.204
Diabetes	126 (22.46%)	10 (32.26%)	136 (22.97%)	0.297
Fatty liver	189 (33.69%)	13 (41.94%)	202 (34.12%)	0.454
Etiology, *n* (%)				
Biliary stones	109 (19.43%)	4 (12.90%)	113 (19.09%)	0.506
Alcohol	20 (3.57%)	3 (9.68%)	23 (3.89%)	0.113
HTG	294 (52.41%)	21 (67.74%)	315 (53.21%)	0.139
Complications, *n* (%)				
APFC	135 (24.06%)	31 (100.00%)	166 (28.04%)	<0.001
ANC	22 (3.92%)	15 (48.38%)	37 (6.25%)	<0.001
PP	2 (0.36%)	3 (9.68%)	5 (0.85%)	<0.001
WON	0 (0.0%)	4 (12.90%)	4 (0.68%)	<0.001
Hospital stay (days)	7 (5)	20 (11)	7 (6)	<0.001
ICU admission, *n* (%)	4 (0.71%)	28 (90.32%)	32 (5.41%)	<0.001
Mortality, *n* (%)	0 (0.0%)	3 (9.68%)	3 (0.51%)	<0.001
Laboratory tests				
WBC (≥18.68 × 10^9^/L)	49 (8.73%)	10 (32.26%)	59 (9.97%)	<0.001
Neutrophils (≥14.34 × 10^9^/L)	84 (14.97%)	12 (38.71%)	96 (16.22%)	<0.001
Lymphocytes (<1.71 × 10^9^/L)	325 (57.93%)	24 (77.42%)	349 (58.95%)	0.05
Monocytes (≥0.75 × 10^9^/L)	148 (26.38%)	12 (38.71%)	160 (27.03%)	0.195
Platelets (≥136.45 × 10^9^/L)	512 (91.27%)	23 (74.19%)	535 (90.37%)	0.006
CRP (≥56.28 mg/L)	139 (24.78%)	26 (83.87%)	165 (27.87%)	<0.001
ALT (≥26.90 U/L)	324 (57.75%)	21 (67.74%)	345 (58.28%)	0.362
AST (≥36.60 U/L)	182 (32.44%)	15 (48.39%)	197 (33.28%)	0.101
Creatinine (≥92.76 μmol/L)	38 (6.774%)	8 (25.806%)	46 (7.770%)	<0.001
BUN (≥6.32 mmol/L)	121 (21.57%)	16 (51.62%)	137 (23.14%)	<0.001
Calcium (<2 mmol/L)	50 (8.92%)	9 (29.03%)	59 (9.97%)	0.002
NLR (≥5.73)	308 (54.90%)	28 (90.32%)	336 (56.76%)	<0.001
mGPS (≥2)	16 (2.85%)	20 (64.52%)	36 (6.08%)	<0.001
SIG (≥4)	15 (2.67%)	20 (64.52%)	35 (5.92%)	<0.001
SII (≥1004.53)	357 (63.64%)	28 (90.32%)	385 (65.03%)	0.005
SIRI (≥4.27)	240 (42.78%)	22 (70.97%)	262 (44.26%)	0.004
PLR (≥144.52)	268 (47.77%)	20 (64.52%)	288 (48.65%)	0.103
dNLR (≥4.36)	262 (46.70%)	27 (87.10%)	289 (48.82%)	<0.001
PNI (<41.22)	51 (9.09%)	22 (70.97%)	73 (12.33%)	<0.001

WBC, white blood cell; HTG, hypertriglyceridemia; CRP, C-reactive protein; BUN, blood urea nitrogen; APFC, acute peripancreatic fluid collection; ANC, acute necrotic collection; PP, pancreatic pseudocyst; WON, walled-off pancreatic necrosis; NLR, neutrophil-to-lymphocyte ratio; mGPS, modified Glasgow prognostic score; SIG, systemic inflammatory grade; SII, systemic immune inflammation index; SIRI, systemic inflammation response index; PLR, platelets-to-lymphocyte ratio; dNLR, derived neutrophil-to-lymphocyte ratio; PNI, prognostic nutritional index.

### SIG, CRP, PNI, and WBC are independent risk factors for SAP

**Figure 2:**
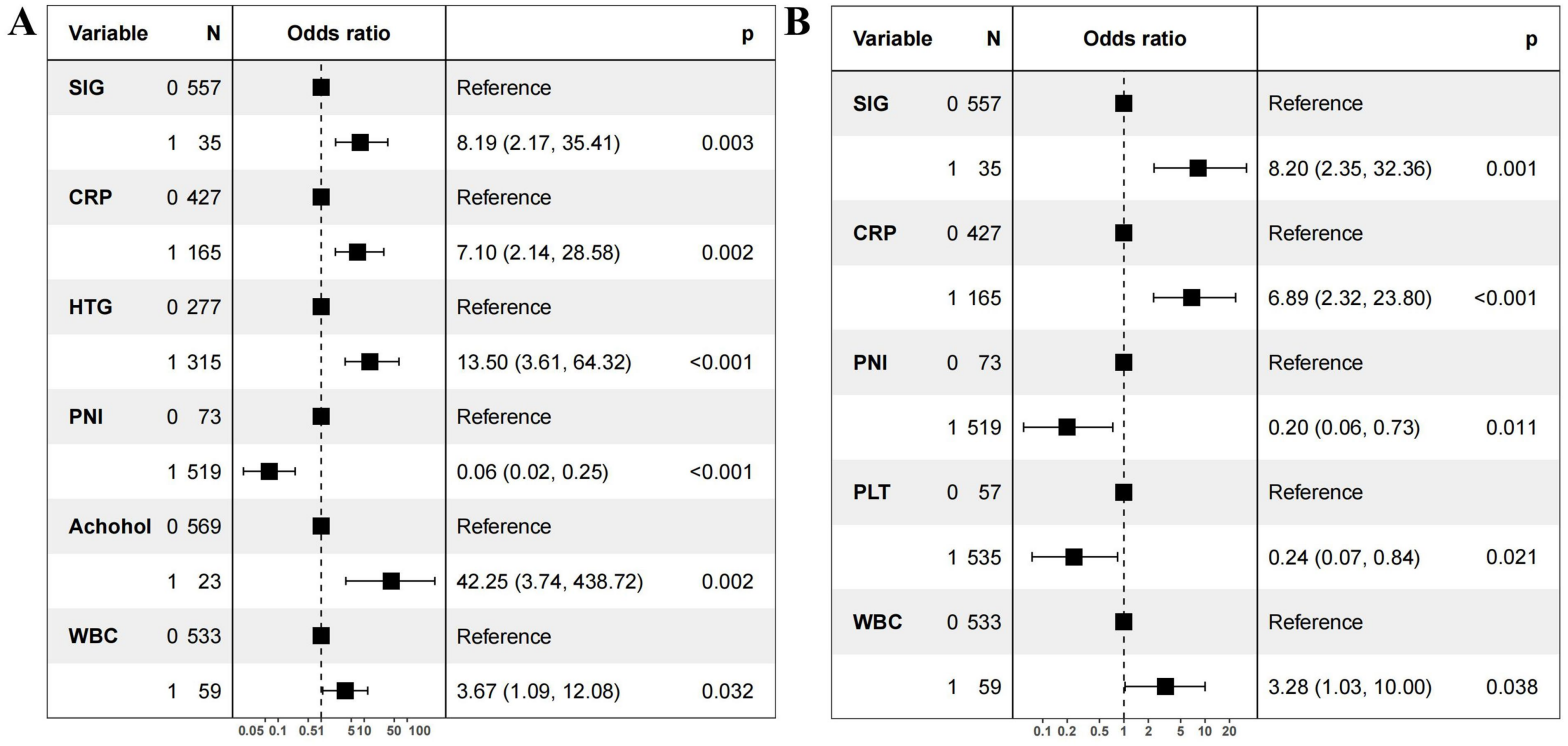
Forest plot of independent risk factors for severe acute pancreatitis. **(A)** Stepwise regression on all variables. **(B)** Stepwise regression on variables with statistical significance (*p* < 0.05) in the univariate analysis. OR, odds ratio; CI, confidence interval. SIG (Systemic Inflammatory Grade) 0: <4; SIG 1: ≥4. CRP (C-reactive protein) 0: <56.28mg/L; CRP 1: ≥56.28mg/L. PNI (prognostic nutritional index) 0: ≥41.22; PNI 1: <41.22. WBC (white blood cell) 0: <18.68×10^9^/L; WBC 1: ≥18.68×10^9^/L. PLT (platelet) 0 (≥136.45×10^9^/L); PLT 1<:136.45×10^9^/L. HTG (hypertriglyceridemia) 0: no; HTG 1: yes.

**Figure 3:**
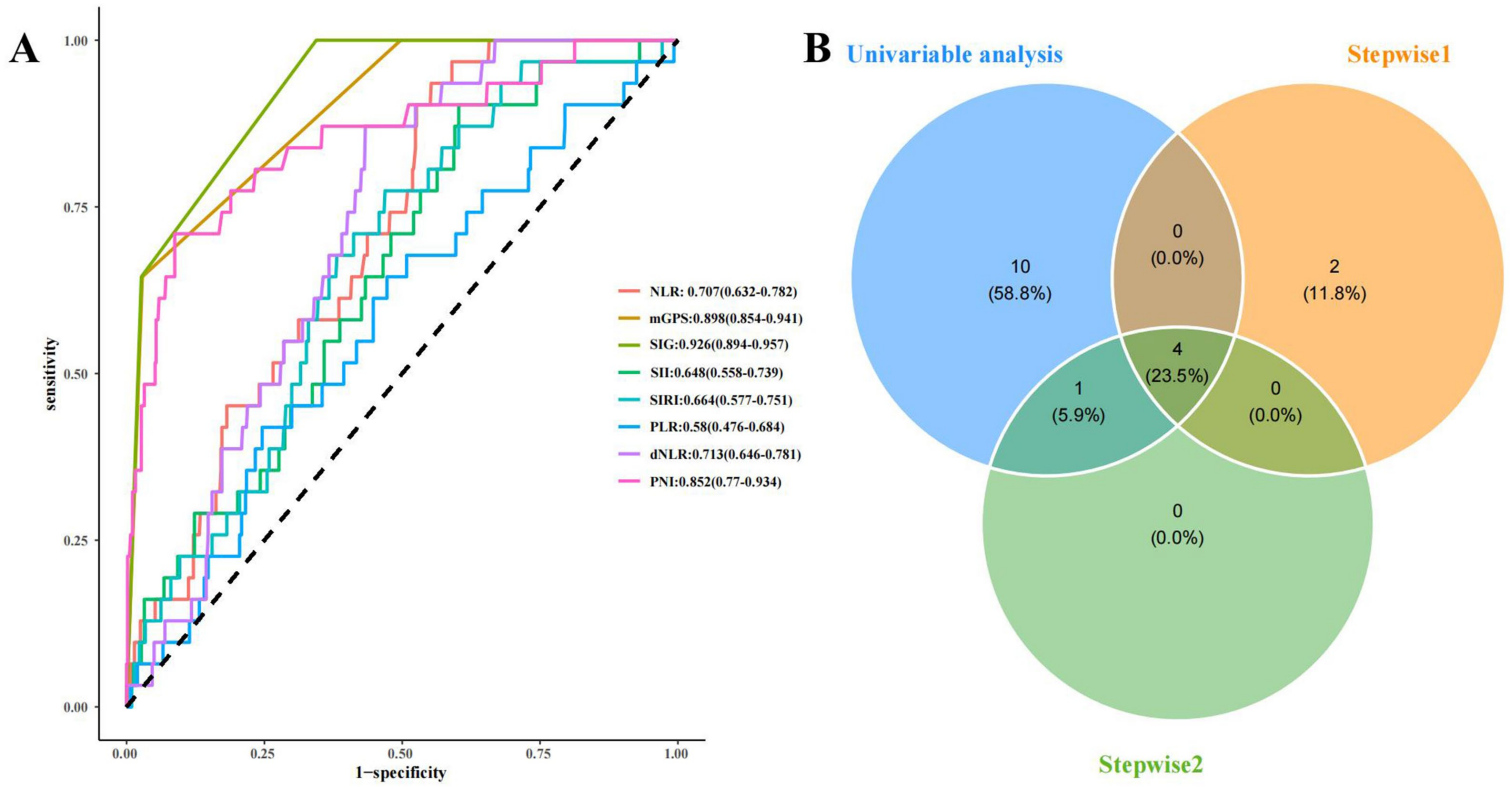
The predictive value of various inflammation scoring systems for SAP and the screening of risk factors for SAP. **(A)** ROC curve of various inflammation scoring systems. **(B)** the screening of risk factors for SAP. NLR, neutrophil-to-lymphocyte ratio; mGPS, modified Glasgow prognostic score; SIG, Systemic Inflammatory Grade; SII, systemic immune inflammation index; SIRI, systemic inflammation response index; PLR, platelets-to-lymphocyte ratio; dNLR, derived neutrophil-to-lymphocyte ratio; PNI, prognostic nutritional index; Stepwise1: the stepwise regression that included all features. Stepwise2: the stepwise regression that included the features with *P*<0.05 from the univariate analysis.

Univariate logistic regression analyses showed that WBC, platelets, neutrophils, lymphocytes, CRP, serum calcium, creatinine, BUN, NLR, mGPS, SIG, SII, SIRI, dNLR, and PNI were significantly related to the occurrence of SAP (Table 2, *p* < 0.05). Two stepwise regression methods were used to further filter the risk factors of SAP (Figure 2). In the Step 1 model, SIG, CRP, HTG, PNI, alcohol, and WBC were selected. In the Step 2 model, SIG, CRP, PNI, PLT, and WBC were identified. At the same time, we examined the predictive value of various inflammation scoring systems for SAP (Figure 3A), and the results showed that SIG had the highest area under the curve (AUC) (0.926, 95% CI 0.894–0.957). We took the intersection of statistically significant variables from univariate analysis, Step 1 model and Step 2 model analysis, resulting in a total of 4 independent risk factors: SIG, CRP, PNI, and WBC (Figure 3B).

**Table 2:** Univariate logistic regression analyses.

Characteristic	OR	95% CI	*p*-value
Gender (male)	0.68	0.33, 1.48	0.315
Age (≥34 years)	3.54	1.04, 22.1	0.087
Smoking	1.25	0.59, 2.58	0.550
Drinking	2.14	0.91, 4.66	0.065
Biliary stones	0.61	0.18, 1.61	0.372
Alcohol	2.90	0.66, 9.11	0.101
HTG	1.91	0.90, 4.30	0.101
Hypertension	1.84	0.78, 4.00	0.137
Diabetes	1.64	0.72, 3.50	0.211
Fatty liver	1.42	0.67, 2.95	0.348
WBC (≥18.68×10^9^/L)	4.98	2.14, 10.9	<0.001
Platelets (<136.45×10^9^/L)	0.28	0.12, 0.69	0.003
Neutrophils (≥14.34×10^9^/L)	3.59	1.64, 7.58	<0.001
Lymphocytes (<1.71×10^9^/L)	0.40	0.16, 0.90	0.037
Monocytes (≥0.75×10^9^/L)	1.76	0.81, 3.68	0.137
CRP (≥56.28mg/L)	15.8	6.45, 47.4	<0.001
Calcium (<2mmol/L)	4.18	1.75, 9.32	<0.001
ALT (≥26.90U/L)	1.54	0.73, 3.46	0.275
AST (≥36.60U/L)	1.95	0.94, 4.05	0.071
Creatinine (≥92.76μmol/L)	4.79	1.90, 11.0	<0.001
BUN (≥6.32mmol/L)	3.88	1.86, 8.15	<0.001
NLR (≥5.73)	7.67	2.68, 32.3	<0.001
mGPS (≥2)	61.9	26.1, 156	<0.001
SIG (≥4)	66.2	27.7, 168	<0.001
SII (≥1004.53)	5.53	1.86, 22.5	0.006
SIRI (≥4.27)	3.27	1.53, 7.60	0.003
PLR (≥144.52)	1.99	0.95, 4.37	0.074
dNLR (≥4.36)	7.70	2.96, 26.3	<0.001
PNI (<41.22)	0.04	0.02, 0.09	<0.001

### Construction of the predictive model and explanation of feature contribution

**Figure 4:**
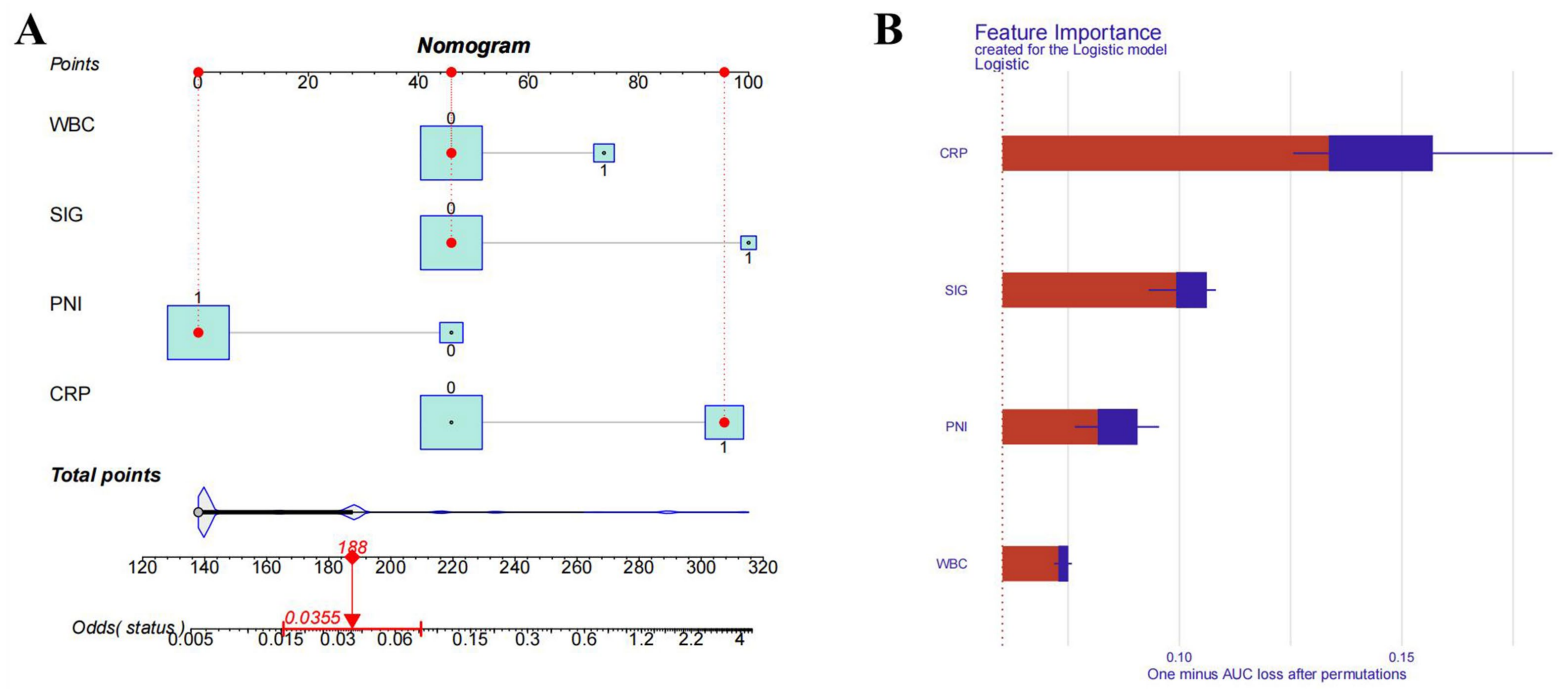
Nomogram model and importance of risk factors. **(A)** Nomogram model. **(B)** Importance of risk factors in the model. CRP, C-reactive protein; SIG, systemic Inflammatory Grade; PNI, prognostic nutritional index; WBC, white blood cell.

By using the four independent influencing factors, which were selected from the previous results, we had established a SAP risk prediction model (the regression equation is: logit (P) = −3.4736 + 2.0392 × SIG + 1.8726 × CRP − 1.7360 × PNI + 1.0473 × WBC), and constructed a visual nomogram (Figure 4A). The importance of feature contributions to the model was explained, and the results showed that CRP was identified as the strongest predictive factor, followed by SIG, PNI, and WBC (Figure 4B).

### The accuracy and stability of the model

**Figure 5:**
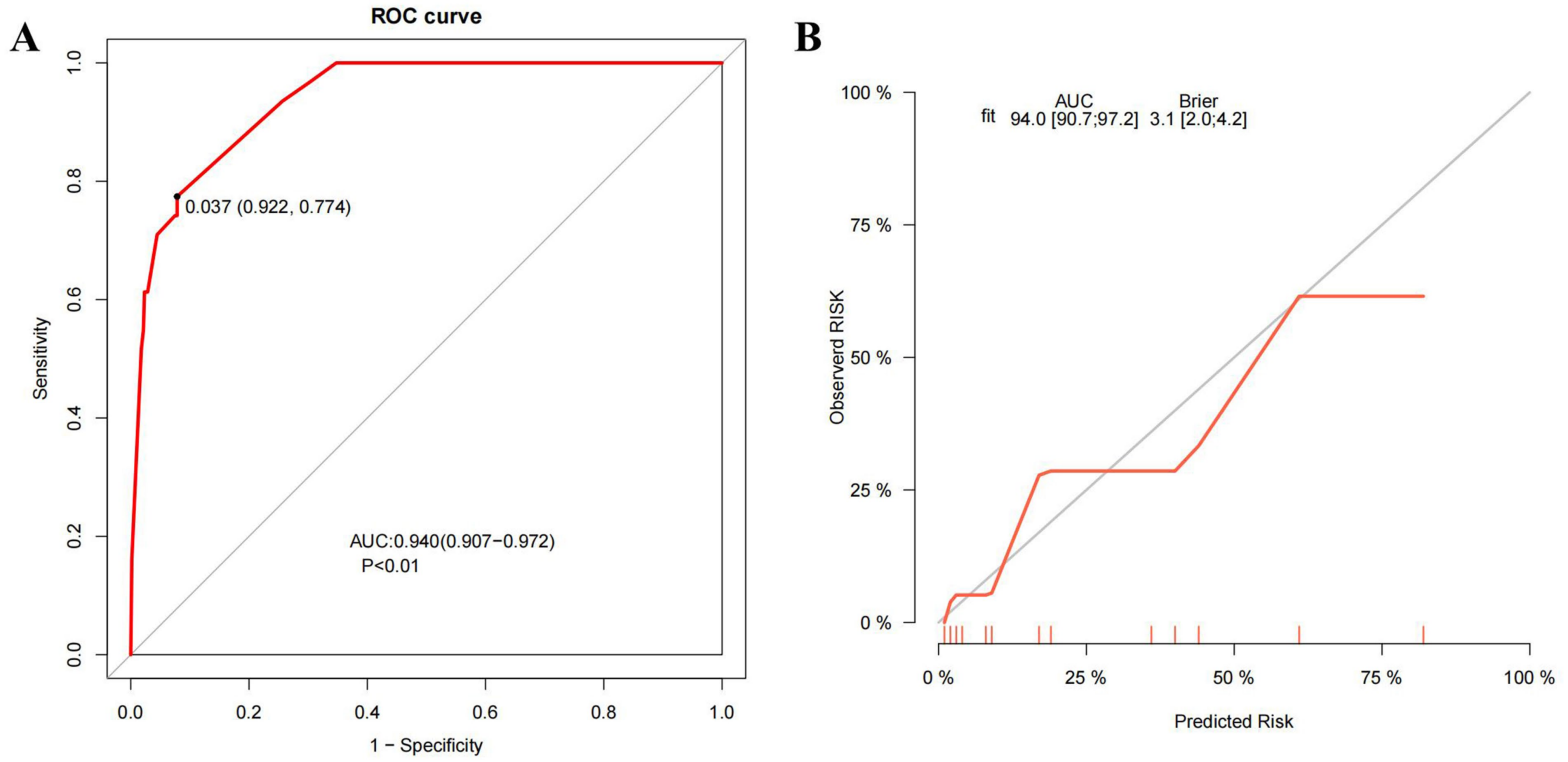
ROC curve and calibration curve of the model. **(A)** The ROC curve of the model. **(B)** The calibration curve of the model. ROC curve, the receiver operating characteristic curve; AUC, the area under the curve; Brier, the Brier Score.

**Figure 6:**
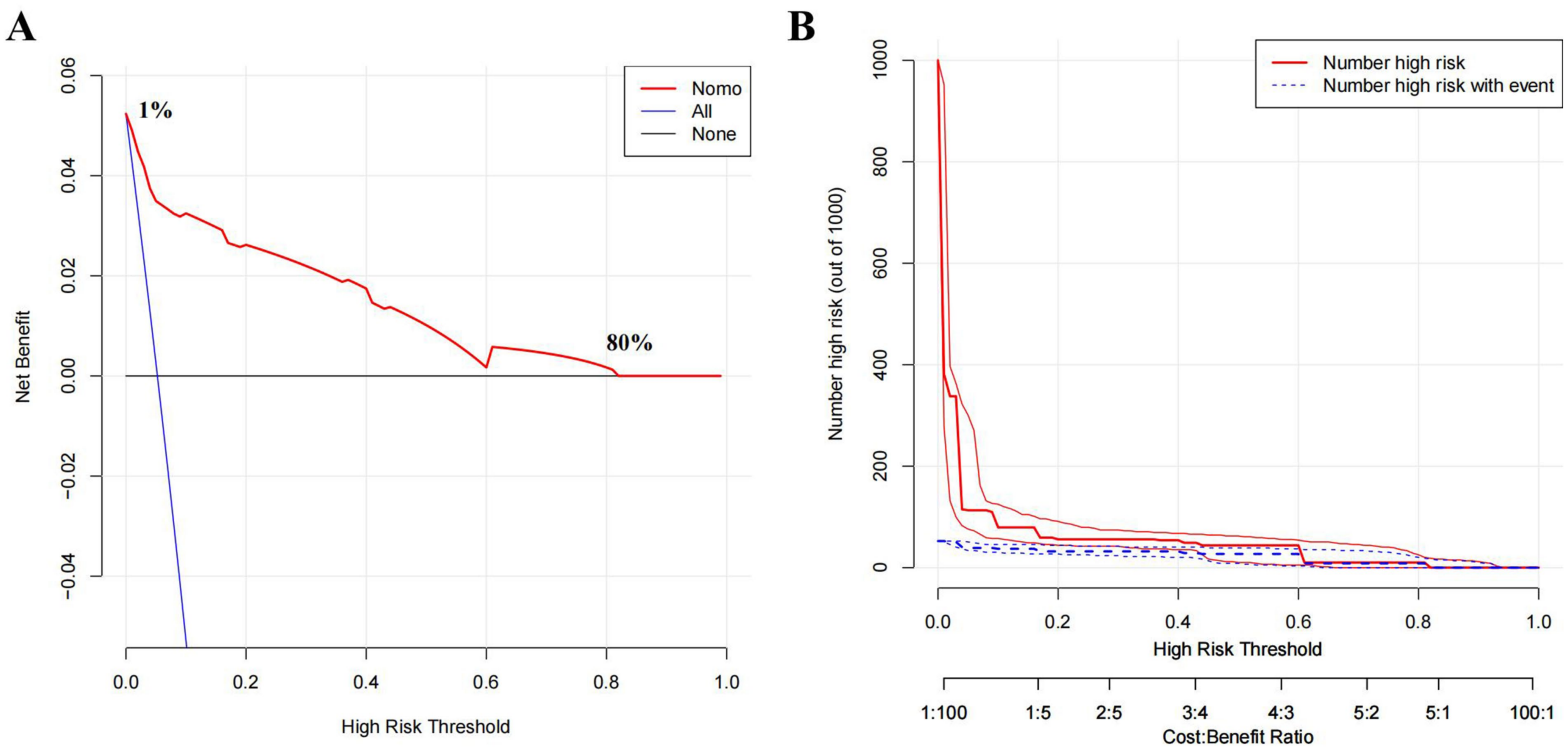
Decision curve and clinical impact curve. **(A)** Decision Curve Analysis (DCA) comparing clinical utility of three strategies. None: Net benefit when no patients are intervened (baseline). All: Net benefit when all patients are intervened, regardless of risk. Nomo (Nomogram): Net benefit when intervention is guided by the predictive model (high-risk patients only).The x-axis represents the high-risk threshold probability for intervention, and the y-axis shows the standardized net benefit. **(B)** Clinical impact curve (CIC). X-axis: High Risk Threshold (probability threshold for classifying high-risk individuals, ranging from 0.0 to 1.0 or as ratios, e.g., 1:100 to 100:1). Y-axis: Number High Risk (out of 1000) (count of individuals classified as high-risk at each threshold or Number High Risk with Event).

The receiver operating characteristic (ROC) curve of the model was plotted (Figure 5A), showing an AUC of 0.940 (95% CI: 0.907–0.972, *p* < 0.01). The cutoff value derived from the maximum Youden index was 0.037, with a sensitivity of 0.774 and a specificity of 0.922. The calibration curve indicated that the model had good degree of calibration (Figure 5B). We assessed the stability of the model using 10-fold cross-validation (Supplementary Figure 1A), and the results showed that both the training datasets (AUC-ROC: 0.940 ± 0.008) and the validation datasets (AUC-ROC: 0.942 ± 0.065) performed well. Additionally, we supplemented the analysis with a confusion matrix based on the aforementioned cutoff (Supplementary Figure 1B). The results of the Hosmer and Lemeshow goodness of fit (GoF) test (X-squared = 1.0349, *p*-value = 0.596) and the Brier score (0.031, 95% CI 0.020–0.042) further demonstrated the accuracy of the model. To further evaluate the value of the model, we plotted the model’s decision curve (DCA) and clinical impact curve (CIC). The results showed that within the broad threshold range of 1–80%, the model provided good predictive value (Figure 6).

## Discussion

Numerous prognostic markers for SAP have been reported. However, their widespread adoption has been hindered by several limitations, and it is still necessary to search for new markers that can effectively predict the severity of AP. In this study, we discovered that SIG was significantly correlated with disease severity during the initial stages of AP. We also analyzed the relationship between AP severity and factors that are involved in the SIG (CRP, mGPS, and NLR). Additionally, we compared SIG with other reported inflammation score systems. Furthermore, we analyzed the feasibility of combining the SIG with other markers in order to predict SAP. Finally, we obtained a SAP prediction model based on CRP, SIG, PNI, and WBC.

CRP, the principal component for calculating mGPS, influences SIG levels. Numerous studies highlighted the high predictive value of serum CRP concentrations for the prognosis of AP, with some researchers positing a threshold of greater than 150 ng/mL at 48 h post-onset as the benchmark for SAP (15). However, this timeframe may not be sufficiently prompt for patients with fast-progressing AP. Several studies described a limited value in using CRP concentration measured at admission or within 48 h for predicting complicated AP (16). In our study, CRP levels within 24 h of admission were positively correlated with AP severity and emerged as an independent risk factor for SAP. Our results are similar to those reported in most of the literature.

No reports have investigated the relationship between mGPS and AP. In our study, mGPS was also not an independent risk factor for SAP. This result may be attributable to the limitation of the mGPS when applied to acute diseases. mGPS is based on CRP and albumin levels, and a serum CRP concentration greater than 10 ng/mL is an important indicator of the prognosis of cancer or chronic disease. However, CRP levels vary widely during the process of acute inflammatory disease, and a cut-off value of 10 ng/mL has obvious limitations.

Most studies consider NLR to be an independent predictor of pancreatitis severity. However, a recent review concluded that NLR possesses only moderate predictive value in predicting AP severity, with inconsistent findings regarding its predictive accuracy reported across various studies (17). In our study, NLR differed in early disease stages among patients with AP of different severities, but it was not identified as independent risk factors for SAP.

Traditional scoring systems such as the Ranson score, APACHE II score, and BISAP score often rely on a series of clinical and laboratory indicators, the acquisition of which may be affected by individual patient differences. For example, the collection time for some data required for the Ranson score needs at least 48 h, which means that in the early stages of AP, physicians may not be able to obtain all the necessary information in a timely manner, thus affecting the timeliness and accuracy of the score (18). Although the APACHE II score can assess the severity of the condition, its complexity and cumbersome calculations also make it difficult to apply quickly in an emergency setting (19). The accuracy of the BISAP score in predicting the mortality risk of biliary pancreatitis is significantly lower than that of other scoring systems (20).

Numerous studies have also explored early and rapid predictive markers, based on simple hematological and biochemical parameters, for SAP, such as NLR, the PNI, SII, and SIRI (4–8). In this study, we also compared SIG with these reported inflammation scoring systems, and the results showed that SIG has a higher predictive value. However, a universally accepted marker has not been established, potentially because of each scoring system’s inherent limitations and their insufficient representation of the disease status when used alone. The integration of multiple indicators may provide new insights into predictive marker development.

In our investigation, a model incorporating SIG, CRP, PNI, and WBC demonstrated robust early warning capabilities for SAP. Besides SIG, CRP, PNI, and WBC all have been reported to be related to the severity of pancreatitis. WBC count is the most commonly used clinical indicator of the body’s inflammatory status, and the early increase of WBC in AP patients is significant for predicting SAP, which provides higher predictive value when combined with other indicators (21). PNI, calculated from serum albumin levels and lymphocyte counts, was initially used to predict the prognosis of cancer patients (22). Studies have found that AP patients with a PNI score >45 have significantly better clinical outcomes than those with lower PNI scores (8).

Theoretically, SIG, derived from mGPS and NLR, might more comprehensively reflect the inflammatory state than CRP, mGPS, or NLR alone. However, CRP contributes the most to the predictive model, followed by SIG. The reason for the lower importance of SIG compared to CRP may be related to the calculation method of SIG. This is mainly because of the limitations of mGPS in its use in acute diseases, which affects the assessment accuracy of SIG. Although mGPS has poor assessment capability for acute inflammatory status, NLR appropriately compensates for this deficiency. Therefore, the predictive value of SIG for SAP is higher than that of mGPS and NLR, but lower than that of CRP.

Despite its contributions, this study had several limitations. First, it was an observational, single-center, retrospective study. The core limitations of retrospective studies include reliance on historical data, which may lead to selection bias and information bias; inadequate adjustment for confounding factors; and the inability to establish a clear temporal relationship between exposure and outcome. Consequently, these factors affect the reliability of causal inferences and limit the generalizability of the study findings. Second, the relatively small sample size of patients with SAP (*n* = 31) might have affected the reliability of our statistical analysis. Finally, the variability in the time lag between illness onset and blood specimen collection might have influenced the laboratory test results.

## Conclusion

SIG holds promise as a potential early prognostic indicator for SAP. Combining CRP, SIG, PNI, and WBC could form an effective early predictive model for SAP.

## Data Availability

The original contributions presented in the study are included in the article/Supplementary material, further inquiries can be directed to the corresponding author.

## Supplementary material

SUPPLEMENTARY FIGURE 1 Ten-fold cross-validation and confusion matrix. **(A)** Ten-fold cross-validation: Curves of AUC values for the training set and validation set plotted in 10-fold cross-validation. **(B)** Confusion matrix: 0 represents Non-SAP, and 1 represents SAP.
