# Supplementary figures and images for: Development and validation of a visual prediction model for severe acute pancreatitis: a retrospective study
Source: Front Med (Lausanne). 2025 Jul 2;12:1564742. doi: 10.3389/fmed.2025.1564742 (PMC12263550; doi:10.3389/fmed.2025.1564742)

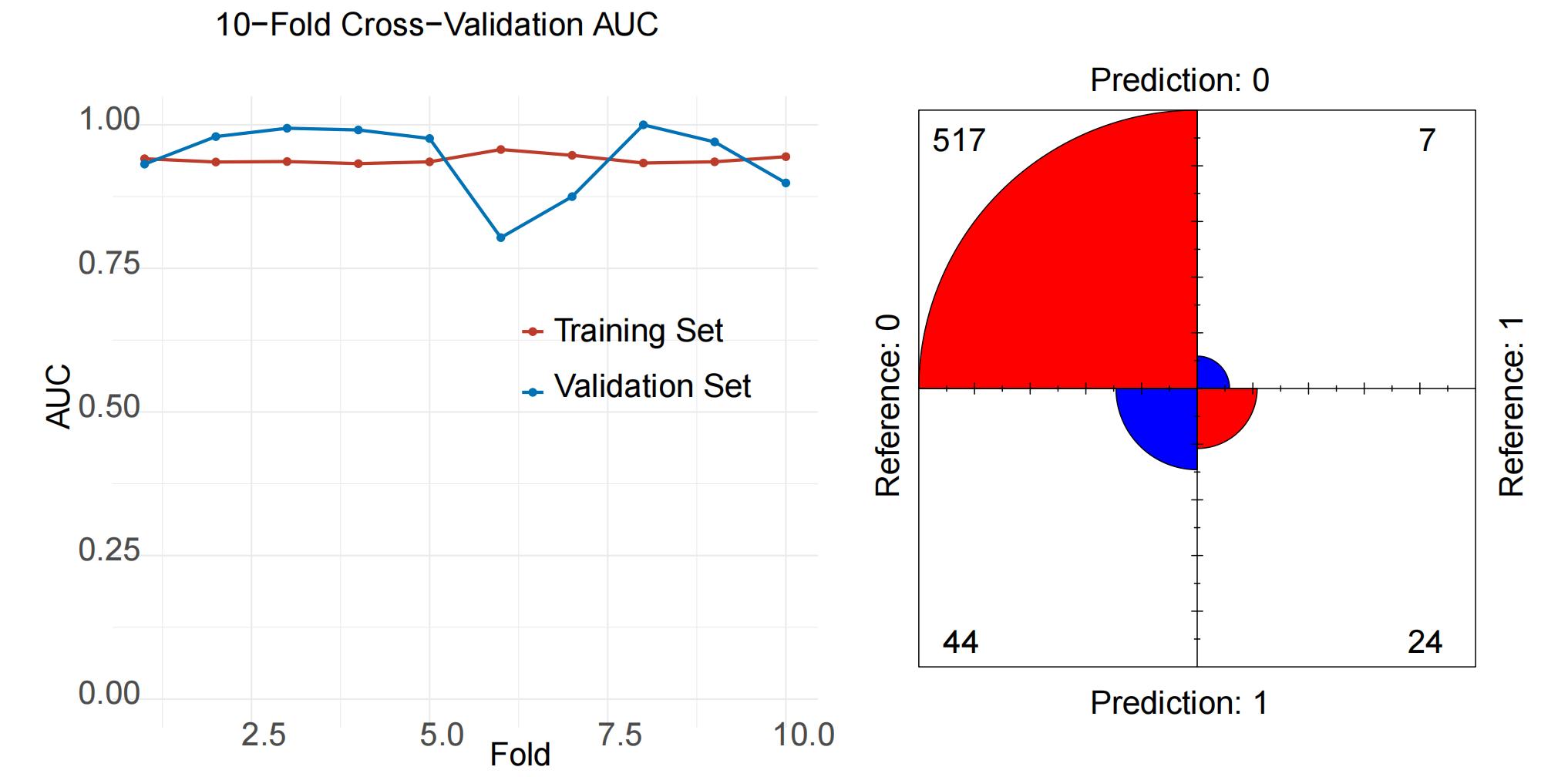

Supplement: SUPPLEMENTARY FIGURE 1 — Ten-fold cross-validation and confusion matrix. (A) Ten-fold cross-validation: Curves of AUC values for the training set and validation set plotted in 10-fold cross-validation. (B) Confusion matrix: 0 represents Non-SAP, and 1 represents SAP. [file Image_1.jpeg]
