# Supplementary material for: Development and validation of a visual prediction model for severe acute pancreatitis: a retrospective study
Source: Front Med (Lausanne). 2025 Jul 2;12:1564742. doi: 10.3389/fmed.2025.1564742 (PMC12263550; doi:10.3389/fmed.2025.1564742)
Supplement: Supplementary file 2 [file Table_1.docx]

**Supplementary Table 1** Measurements of SIG

|  | Score | Criterion |
| --- | --- | --- |
| mGPS | mGPS 0 | CRP≤10 mg/L |
|  | mGPS 1 | C>10 mg/L and albumin≥35 |
|  | mGPS 2 | C>10 mg/L and albumin<35 |
| SIG | SIG 0 | mGPS 0 and NLR < 3 |
|  | SIG 1 | mGPS 0 and NLR 3-5  or  mGPS 1 and NLR < 3 |
|  | SIG 2 | mGPS 0 and NLR > 5  or  mGPS 2 and NLR < 3  or  mGPS 1 and NLR 3-5 |
|  | SIG 3 | mGPS 1 and NLR > 5  or  mGPS 2 and NLR 3-5 |
|  | SIG 4 | mGPS 2 and NLR > 5 |

NLR: neutrophil–to-lymphocyte ratio; mGPS, modified Glasgow prognostic score; SIG, Systemic Inflammatory Grade
