# Supplementary material for: Development and validation of a visual prediction model for severe acute pancreatitis: a retrospective study
Source: Front Med (Lausanne). 2025 Jul 2;12:1564742. doi: 10.3389/fmed.2025.1564742 (PMC12263550; doi:10.3389/fmed.2025.1564742)
Supplement: Supplementary file 3 [file Table_2.docx]

**Supplementary Table 2** Baseline characteristics before converting continuous variables to categorical variables

| **Variables** | **Non-SAP (N=561)** | **SAP (N=31)** | **Total(N=592)** | **P value** |
| --- | --- | --- | --- | --- |
| Demographics |  |  |  |  |
| Age (years) | 43 (35, 55) | 41(35, 53) | 43 (35, 55) | 0.87 |
| Sex (male,%) | 392 (69.88%) | 19 (61.29%) | 411 (69.43%) | 0.418 |
| Smoking, n (%) | 223 (39.75%) | 14 (45.16%) | 237 (40.03%) | 0.682 |
| Drinking, n (%) | 90 (16.04%) | 9 (29.03%) | 99 (16.72%) | 0.101 |
| Comorbidities, n (%) |  |  |  |  |
| Hypertension | 102 (18.18%) | 9 (29.03%) | 111 (18.75%) | 0.204 |
| Diabetes | 126 (22.46%) | 10 (32.26%) | 136 (22.97%) | 0.297 |
| Fatty liver | 189 (33.69%) | 13 (41.94%) | 202 (34.12%) | 0.454 |
| Etiology, n (%) |  |  |  |  |
| Biliary stones | 109 (19.43%) | 4 (12.90%) | 113 (19.09%) | 0.506 |
| Alcohol | 20 (3.57%) | 3 (9.68%) | 23 (3.89%) | 0.113 |
| HTG | 294 (52.41%) | 21 (67.74%) | 315 (53.21%) | 0.139 |
| Complications, n (%) |  |  |  |  |
| APFC | 135 (24.06%) | 31 (100.00%) | 166 (28.04%) | <0.001 |
| ANC | 22 (3.92%) | 15 (48.38%) | 37 (6.25%) | <0.001 |
| PP | 2 (0.36%) | 3 (9.68%) | 5 (0.85%) | <0.001 |
| WON | 0 ( 0.0%) | 4 (12.90%) | 4 (0.68%) | <0.001 |
| Hospital stay (days) | 7 (5) | 20 (11) | 7 (6) | <0.001 |
| ICU admission, n (%) | 4 (0.71%) | 28 (90.32%) | 32 (5.41%) | <0.001 |
| Mortality, n (%) | 0 ( 0.0%) | 3 (9.68%) | 3 (0.51%) | <0.001 |
| Laboratory tests |  |  |  |  |
| WBC, ×10^9^/L | 12.08 (9.10, 15.22) | 12.75 (10.61, 19.34) | 12.15 (9.14, 15.32) | 0.074 |
| Neutrophils, ×10^9^/L | 9.56 (6.75, 12.69) | 10.66 (9.23, 16.84) | 9.66 (6.89, 12.79) | 0.004 |
| Lymphocytes, ×10^9^/L | 1.49 (1.03, 2.10) | 1.28 (0.73, 1.70) | 1.49 (1.01, 2.06) | 0.034 |
| Monocytes, ×10^9^/L | 0.56 (0.41, 0.76) | 0.59 (0.41, 0.90) | 0.56 (0.41, 0.77) | 0.587 |
| Platelets, ×10^9^/L | 217.00 (175.00, 265.00) | 201.00 (137.50, 262.00) | 216.00 (174.50, 265.00) | 0.217 |
| CRP, mg/L | 10.92 (2.64, 54.65) | 192.74 (85.85, 200.00) | 13.52 (2.86, 67.51) | <0.001 |
| ALT, U/L | 30.00 (19.00, 64.000) | 33.00 (22.00, 62.50) | 30.00 (19.00, 65.00) | 0.668 |
| AST, U/L | 26.00 (20.00, 47.00) | 36.000 (25.50, 56.50) | 27.00 (20.00, 48.00) | 0.051 |
| Creatinine, μmol/L | 60.00 (50.40, 71.50) | 60.00 (50.00, 87.50) | 60.00 (50.40, 71.90) | 0.486 |
| BUN, mmol/L | 4.79 (3.86, 5.98) | 6.32 (4.80, 8.60) | 4.83 (3.91, 6.15) | <0.001 |
| Calcium, mmol/L | 2.24 (2.12, 2.33) | 2.11 (1.98, 2.34) | 2.23 (2.11, 2.34) | 0.011 |
| Amylase, U/L | 192.00 (76.00, 573.00) | 391.00(204.00, 796.00) | 199.00 (78.00, 590.50 | 0.018 |
| Lipase, U/L | 280.00 (78.00, 811.00) | 616.00 (241.00, 1403.00) | 288.50 (80.50, 854.00) | 0.005 |
| NLR | 6.22 (3.70, 10.39) | 10.18 (6.33, 15.74) | 6.35 (3.83,10.74) | <0.001 |
| mGPS |  |  |  | <0.001 |
| 0 | 282 (50.27%) | 0 ( 0.00%) | 282 (47.64%) |  |
| 1 | 263 (46.88%) | 11 (35.48%) | 274 (46.28%) |  |
| 2 | 16 (2.85%) | 20 (64.52%) | 36 (6.08%) |  |
| SIG |  |  |  | <0.001 |
| 0 | 71 (12.66%) | 0 ( 0.00%) | 71 (11.99%) |  |
| 1 | 84 (14.97%) | 0 ( 0.00%) | 84 (14.19%) |  |
| 2 | 213 (37.97%) | 0 ( 0.00%) | 213 (35.98%) |  |
| 3 | 178 (31.73%) | 11 (35.48%) | 189 (31.93%) |  |
| 4 | 15 (2.67%) | 20 (64.52%) | 35 (5.91%) |  |
| SII | 1318.15 (775.29, 2314.390) | 1854.55 (1267.97, 3361.76) | 1349.30 (799.19, 2347.09) | 0.005 |
| SIRI | 3.49 (1.78, 6.96) | 5.47 (3.82, 9.03) | 3.58 (1.81, 7.02) | 0.002 |
| PLR | 139.72 (102.70, 206.90) | 163.30 (121.25, 224.98) | 142.05 (103.175;209.400) | 0.134 |
| dNLR | 4.14 (2.74, 6.26) | 5.95 (4.80, 8.35) | 4.28 (2.84, 6.46) | <0.001 |
| PNI | 50.50 (45.75，54.65) | 39.55 (35.25，44.25) | 50.13 (45.18，54.43) | <0.001 |

WBC, white blood cell; HTG, hypertriglyceridemia; CRP, C-reactive protein; BUN, blood urea nitrogen; APFC, acute peripancreatic fluid collection; ANC, acute necrotic collection; PP, pancreatic pseudocyst; WON, walled-off pancreatic necrosis; NLR, neutrophil-to-lymphocyte ratio; mGPS, modified Glasgow prognostic score; SIG, Systemic Inflammatory Grade; SII, systemic immune inflammation inde; SIRI, systemic inflammation response index; PLR, platelets-to-lymphocyte ratio; dNLR, derived neutrophil-to-lymphocyte ratio；PNI, prognostic nutritional index.
